# Secondary Findings from Exome Sequencing of a Greek Cohort

**DOI:** 10.3390/cimb47040272

**Published:** 2025-04-11

**Authors:** Charilaos Kostoulas, Athanasia Sesse, Ioanna Bouba, Spyridon Konitsiotis, Sofia Markoula, Ioannis Georgiou

**Affiliations:** 1 Laboratory of Medical Genetics in Clinical Practice, Faculty of Medicine, School of Health Sciences, University of Ioannina, 45110 Ioannina, Greece; chkost@uoi.gr (C.K.); a.sesse@uoi.gr (A.S.); ibouba@uoi.gr (I.B.); 2 Department of Neurology, Faculty of Medicine, School of Health Sciences, University of Ioannina, 45110 Ioannina, Greece; skonitso@uoi.gr (S.K.); smarkoula@uoi.gr (S.M.)

**Keywords:** secondary findings, exome sequencing, ACMG/AMP guidelines, genetic test

## Abstract

Exome sequencing (ES) is an essential part in clinical diagnosis of hereditary disorders. However, ES can reveal secondary findings (SFs) in medically actionable genes that are not related to the patient’s phenotype. In this study, we performed ES to 280 unrelated individuals of a Greek cohort and calculated the frequency of SFs in 81 ACMG SF v3.2 genes. Variants were classified using the standards and guidelines established by the American College of Medical Genetics and Genomics (ACMG). We identified 12 individuals (4.3%) who carried a pathogenic (P)/likely pathogenic (LP) variant in genes associated with dominant disorders. The variants were found in genes *BRCA1*, *BRCA2*, *MSH6*, *LDLR*, *MYH7*, and *TTN*. Notably, we discovered a P founder variant for the Greek population and one P variant with high prevalence in *BRCA1* gene. Additionally, we observed a high prevalence of P/LP variants in the *LDLR* gene. In conclusion, this is the first study that calculates the prevalence of P/LP variants in the ACMG actionable gene list for SFs in Greece. The results of our study could serve as a guide for the national carrier screening program and may contribute to the precise treatment of certain human disorders.

## 1. Introduction

Over the last two decades, technological advances in Next-Generation Sequencing (NGS) have led to a decline in the sequencing cost and an increase in the amount of sequencing data produced. Exome sequencing (ES) and genome sequencing (GS) have become crucial for research purposes and clinical diagnosis. ES is particularly used to identify variants which cause complex and rare hereditary disorders. ES produces a huge number of variants, some of which are associated with the disorder for which the patient was referred [1]. However, with ES, variants called secondary findings (SFs) or incidental findings (IFs), in medically actionable genes unrelated to the patient’s disorder, can also be detected.

The American College of Medical Genetics and Genomics (ACMG) has proposed recommendations for reporting SFs in clinical ES or GS. Initially, in 2013, the ACMG released a list of 56 genes, known to be medically actionable, associated with 27 medical conditions [2]. In 2015, the list was updated to 59 genes and 27 conditions in ACMG SF v.2.0 [3]. The last versions of the ACMG lists are listed as ACMG SF v.3.0, ACMG CF v.3.1 and ACMG SF v.3.2, which contain 73 genes, 78 genes, and 81 genes, respectively [4,5,6]. These genes are associated with cardiovascular, cancer, metabolic, and miscellaneous disorders.

Furthermore, ACMG and the Association for Molecular Pathology (AMP) released 28 criteria for the interpretation of sequence variants. These criteria are applicable to many genes and disorders with different hereditary patterns. According to the ACMG guidelines, two pathogenic (P) or likely pathogenic (LP) variants in ACMG SF v3.2 genes associated with autosomal recessive (AR) inheritance, one variant in genes associated with dominant inheritance and at least one variant in ACMG SF v3.2 genes associated with X-linked (XL) disorders should be reported [6].

In this study, we calculated the frequencies of SFs in medically actionable ACMG v3.2 genes, as well as the carrier rate and the at-risk couple rates for genes associated with recessive disorders. The SFs were drawn from a regional Greek cohort of 280 unrelated patients, who were referred for rare disorder diagnosis. The aim of our study was to identify—through ES—the frequency of SFs of 81 genes and to determine the most common P/LP variants in the Greek population. To the best of our knowledge, this is the first retrospective study for SFs conducted in Greece.

## 2. Materials and Methods

**Figure 1:**
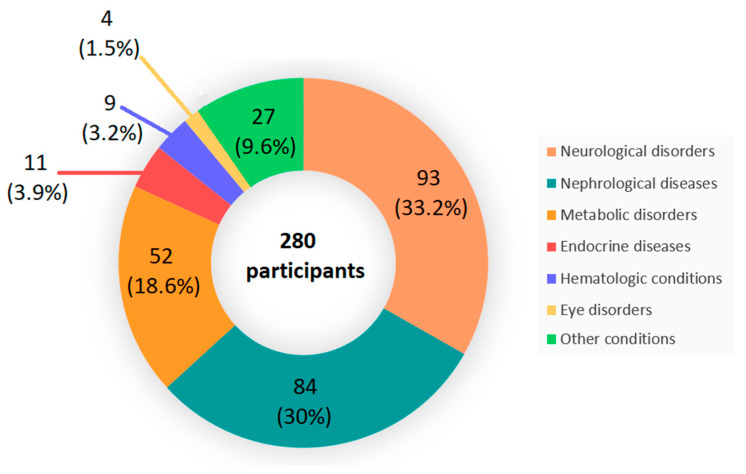
Distribution of primary indications for referral in 280 individuals enrolled in the study.

A total of 280 individuals, initially referred for rare disorder testing, were enrolled in this study. Clinical and demographic information was collected and written informed consent was obtained from all participants or their legal guardians. The primary indications for referral included neurological disorders (33.2%), nephrological diseases (30%), metabolic disorders (18.6%), endocrine disorders (3.9%), hematologic conditions (3.2%), eye disorders (1.5%), cardiovascular diseases (0.7%), and other conditions (8.9%) (Figure 1).

Peripheral blood samples were collected from each participant and genomic DNA was extracted from EDTA blood leukocytes using the NucleoSpin Blood kit, Macherey-Nagel, Dueren, Germany. Whole-Exome Sequencing (WES) was performed using the DNA Prep Exome2.0 Plus Enrichment and Mitochondrial DNA, Illumina, following the manufacturer’s recommendations. Prepared DNA libraries were sequenced on NextSeq 1000, Illumina, San Diego, CA, USA. WES data quality control standards included a mean depth of coverage >100×, with >98% regions at 20×. Variant calling was performed using the Genome Analysis Toolkit (GATK) version 4.5.0.0 and selected variants were filtered using a minor allele frequency (MAF) of <1%.

**Figure 2:**
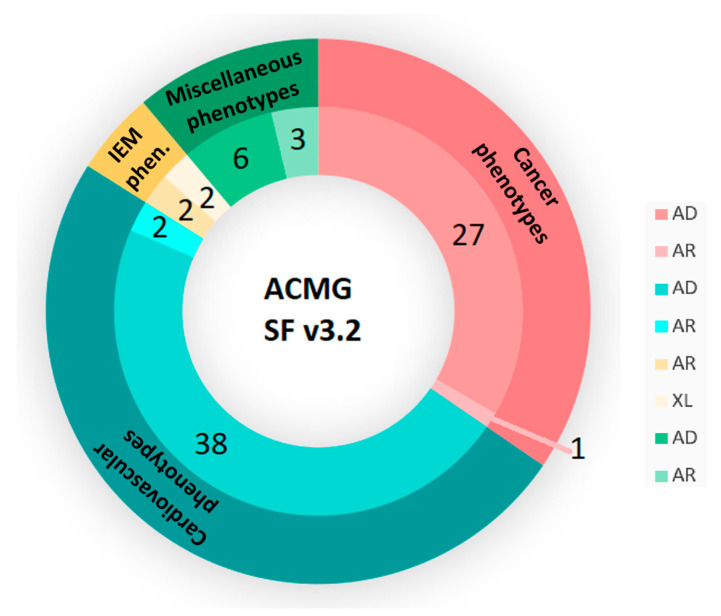
Distribution of genes from the ACMG’s SF v3.2 list by phenotype and inheritance pattern.

**Figure 3:**
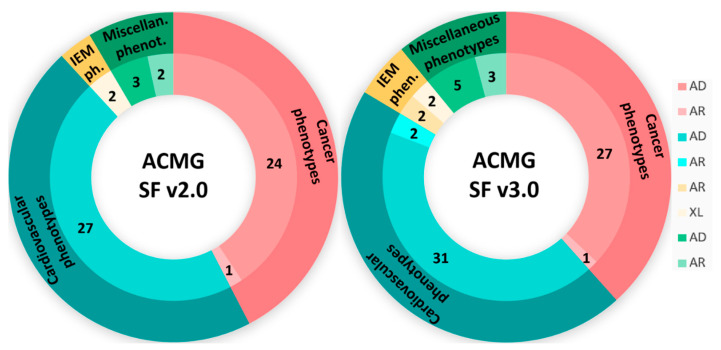
Distribution of genes from the ACMG’s SF v2.0 (**left**) and v3.0 (**right**) list by phenotype and inheritance pattern.

Variant interpretation was carried out using the Franklin by Genoox version 82 software and was limited to the 81 clinically actionable genes recommended by the ACMG in the SF v3.2 list (Supplementary Table S1) [6]. The list included 28 genes related to cancer phenotypes (27 autosomal dominant (AD) and 1 AR), 40 genes associated with cardiovascular phenotypes (38 AD and 2 AR), 4 genes associated with inborn errors of metabolism (IEM) phenotypes (2 AR and 2 XL), and 9 genes related to miscellaneous phenotypes (6 AD and 3 AR) (Figure 2). The same analysis was also performed using the ACMG SF v2.0 (59 genes—Supplementary Table S2, Figure 3) and v3.0 (73 genes—Supplementary Table S3, Figure 3) lists in order to assess the yield across different versions [3,4]. It is noted that none of the individuals have a genetic diagnosis associated with any of these genes.

Variants were classified using the standards and guidelines established by the ACMG, and only P or LP variants were included in the analysis [7]. In particular, variants that were previously reported in genomic databases (i.e., ClinVar, ClinGen, LOVD) or the literature and classified as P/LP, as well as novel nonsense, frameshift, and splice site variants classified as P/LP and predicted to cause the disorder or variants with a pLoF (predicted loss-of-function) annotation in genes, where loss-of-function is a known cause of disease, were considered medically actionable and included in the study.

For AR or XL conditions, at-risk couple rates were also calculated. The at-risk couple rate is the probability that both partners are carriers of a P or LP variant in the same gene (AR conditions), or the female is carrier of a P or LP variant (XL conditions). For AR conditions, the at-risk couple rate is defined as the gene’s (or condition’s) carrier rate squared, while for XL conditions it is defined as the gene (condition) carrier rate [8,9].

## 3. Results

### 3.1. Population Demographics

**Figure 4:**
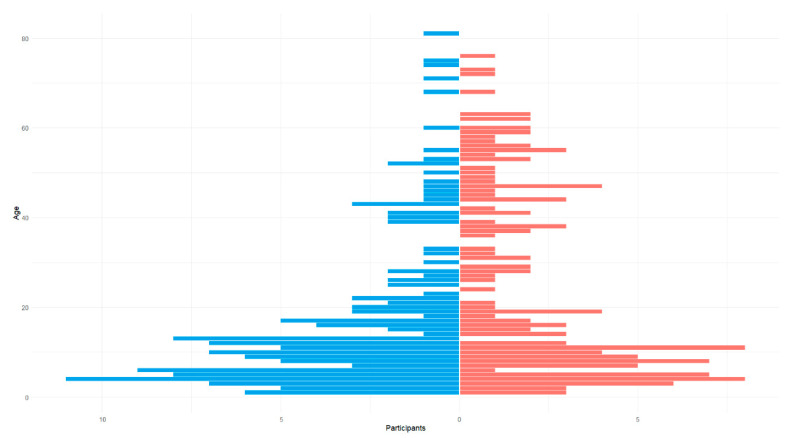
Age and sex breakdown of our cohort. (Blue: male, Pink: female).

A total of 280 individuals participated in the study; 169 (60%) were children and adolescents (<18 years old), while 111 (40%) were over the age of 18 at the time of testing. The mean ± SD age was 21.3 ± 19.6 years. Amongst those individuals, 133 (47.5%) were females and 147 (52.5%) were males (Figure 4). According to the demographic characteristics, the cohort consisted exclusively of individuals from a particular Greek region (northwestern Greece—Epirus).

### 3.2. SFs Analysis

**Figure 5:**
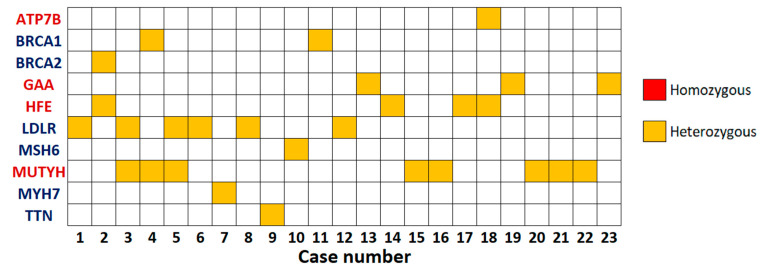
Distribution of the P/LP variants in the 23 positive participants. None of the individuals carried homozygous or compound heterozygous recessive disease alleles. (Genes in blue: AD, genes in red: AR).

A total of 10,258 non-silent variants (single nucleotide variants—SNVs, and INDELs) were detected in the 81 ACMG-recommended genes in WES of 280 individuals [6]. For each participant, there were on average 36.6 non-silent variants. Following the ACMG variant classification standards and guidelines, 28 variants were classified as P or LP in 23 individuals (8.2%) when 81 gene approach was used (Figure 5, Supplementary Table S4) [7]. Specifically, those variants were either reported previously in the ClinVar database as causative variants of associated diseases or were novel but were classified as P/LP by ACMG criteria and could cause premature termination of the codon in a loss-of-function mechanism gene (including nonsense, frameshift, and canonical splice site variants). In addition, percentages of SF in the 59 ACMG genes (v2.0) were also calculated, resulting in a yield of 6.8% (19 carriers, 24 variants) [3]. When the percentage of SF in 73 genes (v3.0) was calculated, a yield of 8.2% was obtained (23 individuals, 28 variants) [4]. Number of carriers and variants in 59, 73, and 81 ACMG genes are presented in Table 1.

#### 3.2.1. Dominant Actionable Genes

Amongst the 280 individuals who were screened, 12 (4.3%) had one medically actionable variant. Furthermore, 9 of the different variants were related to 6 out of the 71 dominant actionable genes (Table 2). Two of those variants were detected in more than one individual.

Eight variants in three genes associated with cardiovascular phenotypes were found in eight individuals, thus constituting 66.7% of the individuals with SFs in dominant actionable genes and 2.9% of the entire study group. There were 5 men, representing 3.4% of the 147 males in the study, and 3 were women, representing 2.3% of the 133 females. The most frequent gene was *LDLR* (six carriers), which is associated with familial hypercholesterolemia [10,11]. Three different variants were identified in *LDLR*, with the c.858C>A (p.Ser286Arg) variant detected in three participants, while the c.1775G>A (p.Gly592Glu) variant was detected in two participants. Furthermore, c.2609G>T p.(Arg870Leu) and c.52903C>T p.(Arg17635*) were identified in *MYH7* (associated with hypertrophic cardiomyopathy) and *TTN* (associated with dilated cardiomyopathys), respectively (Table 2) [12,13,14,15].

Variants related to cancer phenotypes were detected in four participants, thus constituting 33.3% of the individuals with SFs in dominant actionable genes and 1.4% of the entire cohort. Three participants out of five were men, representing 2.0% of the males in this study, while one was female (0.8% of the females). The most frequent gene was *BRCA1* with two variants in two individuals (one male and one female) (Table 2). This gene is associated with increased susceptibility to breast and ovarian cancer [16,17,18]. Medically actionable variants were also identified in two other cancer-related genes, including *BRCA2* and *MSH6*, which are associated with hereditary breast and/or ovarian cancer and Lynch syndrome, respectively (Table 1) [16,17,18,19,20].

None of the 280 participants carried a variant of any dominant actionable gene related to IEMs or miscellaneous phenotypes.

#### 3.2.2. Recessive Actionable Genes

In addition to dominant disease alleles, 15 individuals (5.4%) were also identified to be carriers of at least one disease allele in four of the eight recessive actionable genes, with 1 participant carrying two alleles for two recessive disorders. A total of 16 ACMG SF variants, of which 5 were unique, were detected when the 81 gene approach was used (Table 3). Four carriers of a heterozygous recessive high-risk disease allele also had a dominant high-risk disease allele (Supplementary Table S4).

P/LP variants in a gene predisposed to hereditary cancer were most frequently observed. More specifically, the c.1103G>A (p.Gly368Asp) variant in the *MUTYH* gene was found in eight individuals, constituting 53.3% of the subjects with SFs in recessive medical actionable genes and 2.9% of the entire cohort (Table 3). There were 5 female carriers, representing 3.8% of the 133 women in the study, and 3 were male, representing 2.0% of the 147 men. *MUTYH* is related to MUTYH-associated polyposis (MAP) [21,22].

Five variants in two genes (*HFE*, *ATP7B*) in the miscellaneous phenotype category were identified in four individuals (26.7% of the individuals with SFs in recessive actionable genes, 1.4% of the entire study group—two male, two female). Two of those variants were unique (Table 3). The c.845G>A (p.Cys282Tyr) variant in the *HFE* gene, responsible for hereditary hemochromatosis, was the most common (4 carriers), while the c.2304dup (p.Met769Hisfs*26) variant in the *ATP7B* gene was identified in one individual [23,24]. The *ATP7B* gene is associated with Wilson disease [25,26]. In addition, there was one participant carrying two variants for two different miscellaneous recessive disease-associated genes. As a result, *HFE* c.8445G>A (p.Cys282Tyr) and *ATP7B* c.2304dup (p.Met769Hisfs*26) variants were detected in the same case (Supplementary Table S4).

The medically actionable variants associated with IEM phenotypes were detected in three individuals, representing 20.0% of the individuals with SFs in recessive genes and 1.1% of our cohort. Amongst these three individuals, there were two men and one woman. In total, two different variants were identified in the *GAA* gene: c.-32-13T>G in two individuals, and c.1465G>A (p.Asp489Asn) in one individual. The *GAA* gene is associated with Pompe disease (Table 3) [27,28].

Finally, the at-risk couple rates as well as the carrier rates of the four recessive actionable genes that were detected in our cohort are listed in Table 4.

It is noted that none of the 15 individuals—with variants in genes associated with AR conditions—carried homozygous or compound heterozygous recessive disease alleles. According to the ACMG guidelines for reporting SFs, a single variant in genes linked to recessively inherited phenotypes does not meet the criteria for reporting [6].

## 4. Discussion

In this study, we performed ES to investigate the frequency of genetic variants in the ACMG actionable gene list v3.2 in a patient cohort in Northwestern Greece [6]. Amongst 280 patients, who were referred for a genetic disorder other than the ones included in the ACMG disorder list, we identified 23 individuals carrying at least one variant of the 81 ACMG SF v3.2 recommended genes. The carrier frequency of all the variants was 8.2% in the ACMG gene list. Also, in our study, the prevalence of SFs in the ACMG SF v2.0 and v3.0 gene lists was 6.8% and 8.2%, respectively [3,4].

Furthermore, in our cohort, 12 individuals were carriers of one P or LP variant in one of the 71 medically actionable genes associated with a dominant inherited disorder, and no patient carried two variants within a gene associated with recessive inherited disorder. In our study, we obtained a yield of 4.3%, which is consistent with other studies that estimated a carrier frequency range of 2.5 to 5% [29,30,31]. A Serbian cohort study of 443 participants reported a frequency of 3.8% in 81 ACMG SF v3.2 actionable genes [32]. Also, a study from China reported a carrier frequency of 5.3% and a study for the Icelandic population found a frequency of 4% in 73 ACMG SF v3.0 genes [33,34].

The findings of our study showed common variations in the Mediterranean population. More specifically, a study on Italians found the c.1187G>A p.(Gly396Asp) and c.1775G>A p.(Gly592Glu) variants in the *MUTYH* and *LDLR* genes, respectively, at a high frequency, similar to what was revealed in our study [35]. As is well known from previous studies on Mediterranean populations, many variants of thalassemia came from Greece and especially from Northwestern Greece [36,37].

However, our results are not in line with other studies. Two studies involving 21,915 and 1303 individuals reported a carrier frequency of 2.54% and 2.5%, respectively, in the 56 ACMG SF v2.0 gene list [38,39]. The major reason for these differences is the use of different versions of the ACMG gene lists, which leads to disparities in the number of genes analyzed. The wide range of the observed prevalence between the studies is very common and can depend on several factors, such as the NGS technology used as well as the classification of assessed variants. Finally, another reason for the difference in the observed prevalence in our study—in comparison to other studies—is the smaller number of participants in our research versus previous ones [39].

In our study, all participants opted to receive SFs as part of their WES analysis. This decision reflects an active choice to receive additional genetic information in line with the ACMG guidelines for reporting SFs. It is worth noting that other studies, such as one by Brunfeldt et al., have explored the decision-making process regarding SFs, specifically examining factors like sex, age, and country. These studies provide valuable insights into how demographic variables may influence patients’ and families’ decisions to opt in for secondary findings [40]. Additionally, in the same study, it was reported that out of patients who opted in, a SF was detected in 2.7% of the cases.

In our study, we identified eight variants in genes causing cardiac disorders. One individual was a carrier (1/280) of the missense c.2609G>T p.(Arg870Leu) variant in the *MYH7* gene, which has been reported as a P variant for hypertrophic cardiomyopathy. Hypertrophic cardiomyopathy is a common disorder, with its frequency in the general population estimated to be between one in 500 and one in 3000 [41]. Furthermore, in the *TTN* gene, we included only the variant c.52903C>T p.(Arg17635*), as the ACMG guidelines recommend that only frameshift and nonsense variants should be reported in the *TTN* gene.

In our sample, we found six individuals with familial hypercholesterolemia carrying a P or LP variant in the *LDLR* gene. The large number of individuals with familial hypercholesterolemia is common in the Northwestern Greece region and this could explain the high prevalence of pathogenic variants in *LDLR* observed in our study. The three variants that have been identified are common in Northwestern Greece and show a regional distribution [42,43]. More specifically, the two most common variants, c.858C>A p.(Ser286Arg) and c.1775G>A p.(Gly592Glu), were found in three and two individuals, respectively, and may be considered founder mutations. Furthermore, familial hypercholesterolemia is often underdiagnosed, which may contribute to an underestimated prevalence [44,45,46].

Additionally, we identified four individuals who carried variants in cancer predisposition genes. More specifically, the following variants have been identified: c.5497G>A p.(Val1833Met) and c.3700_3704del p.(Val1234Glnfs*8)—which are a well-known founder variant and P variant with high prevalence, respectively, in the Greek population—in the *BRCA1* gene in two individuals; c.8169T>A p.(Asp2723Glu) in the *BRCA2* gene and c.2653A>T p.(Lys885*) in the *MSH6* gene in the other two individuals [47,48]. The two *BRCA1* variants should be included in a potential national carrier screening program for breast and ovarian cancer, as early detection of breast cancer may lead to decreased morbidity with improved cancer outcomes [49].

Furthermore, in our study, we identified that 5.4% of the patients in our cohort were carriers of at least one P/LP variant in the *ATP7B*, *GAA*, *HFE*, and *MUTYH* genes, which are associated with recessive disorders. In the *GAA* gene, we identified three individuals who were carriers of two variants. In the *HFE* gene, four individuals were carriers of one variant while in the *MUTYH* gene, eight individuals were found to be carriers of one variant. Our results suggest that the presence of two specific variants in the *MUTYH* and *HFE* genes could be characterized as founder mutations for Northwestern Greece. However, according to the ACMG guidelines, a heterozygous variant in a recessive-associated gene should not be reported, it might be beneficial to consider the high carrier frequency of P/LP variants and the high at-risk couple rate, especially in specific population groups (such as Northwestern Greece) with notable susceptibility. The high prevalence of carrier status for these four genes could be very valuable for specific populations with a high rate of consanguinity.

The results of our study indicate that certain disorders—such as familial hypercholesterolemia—occur more frequently in Northwestern Greece and they are indeed underdiagnosed. Genetic testing should be conducted even in patients classified as having “unlikely” or “possible” familial hypercholesterolemia, based on the Dutch criteria, as familial hypercholesterolemia could not be ruled out in these cases [50]. Additionally, cascade screening and/or reverse cascade screening should be integrated to improve detection and diagnosis [51].

In summary, this is the first study that estimates the prevalence of SF variants in Greece based on the ACMG SF v3.2 gene list. We identified the prevalence of 4.3% of patients who carry a P or LP variant in a medically actionable gene and who have an increased risk of developing a severe genetic disorder. The results of our project could serve as a guide for the national carrier screening program, which is already implemented in Greece for specific disorders. The data may contribute to the prevention and precise treatment of certain human disorders.

## Figures and Tables

**Table 1:** Number of carriers and variants in 59, 73, and 81 ACMG genes.

	ACMG SF v2.0			ACMG SF v3.0			ACMG SF v3.2		
	AD	AR	XL	AD	AR	XL	AD	AR	XL
No. of carriers	11	12	0	12	15	0	12	15	0
No. of variants	11	13	0	12	16	0	12	16	0

**Table 2:** P/LP variants in AD ACMG-recommended genes. (He: Heterozygous).

Gene	Condition	Transcript	Variant	dbSNP ID	ACMG Classification	Zygosity	Number of Carriers
*BRCA1*	Hereditary breast and/or ovarian cancer	NM_007294.4	c.3700_3704del p.(Val1234Glnfs*8)	rs80357609	P (PVS1, PP5, PM2)	He	1
*BRCA1*	Hereditary breast and/or ovarian cancer	NM_007294.4	c.5497G>A p.(Val1833Met)	rs80357268	P (PP5, PM1, PM5, PP3, PM2)	He	1
*BRCA2*	Hereditary breast and/or ovarian cancer	NM_000059.4	c.8169T>A p.(Asp2723Glu)	rs1060502432	P (PM1, PM5, PP3, PM2, PP5)	He	1
*LDLR*	Familial hypercholesterolemia	NM_000527.5	c.81C>G p.(Cys27Trp)	rs2228671	P (PP5, PP3, PM1, PM5)	He	1
*LDLR*	Familial hypercholesterolemia	NM_000527.5	c.858C>A p.(Ser286Arg)	rs140241383	P (PP5, PS1, PM1, PP3)	He	3
*LDLR*	Familial hypercholesterolemia	NM_000527.5	c.1775G>A p.(Gly592Glu)	rs137929307	P (PS3, PM5, PP3, PM1, PP5)	He	2
*MSH6*	Lynch syndrome (Hereditary Nonpolyposis Colorectal Cancer; HNPCC)	NM_000179.3	c.2653A>T p.(Lys885*)	rs587782593	P (PVS1, PP5, PM2)	He	1
*MYH7*	Hypertrophic cardiomyopathy	NM_000257.4	c.2609G>T p.(Arg870Leu)	rs36211715	P (PM1, PM5, PP3, PP5, PM2)	He	1
*TTN*	Dilated cardiomyopathy	NM_001267550.2	c.52903C>T p.(Arg17635*)	rs2154197219	P (PVS1, PP5, PM2)	He	1

**Table 3:** P/LP variants in AR ACMG-recommended genes. (He: Heterozygous).

Gene	Condition	Transcript	Variant	dbSNP ID	ACMG Classification	Zygosity	Number of Carriers
*ATP7B*	Wilson disease	NM_000053.4	c.2304dup p.(Met769Hisfs*26)	rs137853287	P (PVS1, PP5, PM2)	He	1
*GAA*	Pompe disease	NM_000152.5	c.-32-13T>G p.?	rs386834236	P (PVS1, PM3, PP4)	He	2
*GAA*	Pompe disease	NM_000152.5	c.1465G>A p.(Asp489Asn)	rs398123169	P (PP5, PM5, PP3, PM1, PM2)	He	1
*HFE*	Hereditary hemochromatosis	NM_000410.4	c.845G>A p.(Cys282Tyr)	rs1800562	P (PS3, PS1, PP5, BP1)	He	4
*MUTYH*	MUTYH-associated polyposis (MAP)	NM_001048174.2/ NM_001128425.2	c.1103G>A p.(Gly368Asp)/c.1187G>A p.(Gly396Asp)	rs36053993	P (PP5, PP3, PS3, PM5, PP2)	He	8

**Table 4:** Gene carrier rates and at-risk couple rates of recessive genes.

Gene	Number of Carriers	Gene Carrier Rate	At-Risk Couple Rate
*ATP7B*	1	0.0036	0.000013
*GAA*	3	0.0107	0.000115
*HFE*	4	0.0143	0.000204
*MUTYH*	8	0.0286	0.000818

## Data Availability

The original contributions presented in this study are included in the article/Supplementary Materials. Further inquiries can be directed to the corresponding author.

## Supplementary Materials

The following supporting information can be downloaded at: https://www.mdpi.com/article/10.3390/cimb47040272/s1.

## Abbreviations

The following abbreviations are used in this manuscript:

ACMG	American College of Medical Genetics and Genomics
AD	Autosomal Dominant
AMP	Association for Molecular Pathology
AR	Autosomal Recessive
ES	Exome Sequencing
GATK	Genome Analysis Toolkit
GS	Genome Sequencing
IEM	Inborn Errors of Metabolism
IF	Incidental Finding
LP	Likely Pathogenic
MAF	Minor Allele Frequency
NGS	Next-Generation Sequencing
P	Pathogenic
pLoF	Predicted Loss-of-Function
SF	Secondary Finding
SNV	Single Nucleotide Variant
WES	Whole-Exome Sequencing
XL	X-Linked
